# Dealing Wildlife Offences in India: Role of the Hair as Physical Evidence

**DOI:** 10.4103/0974-7753.51928

**Published:** 2009

**Authors:** Vivek Sahajpal, SP Goyal, Kumudbala Singh, Vinod Thakur

**Affiliations:** Wildlife Forensic Laboratory, Noida, India; 1 Wildlife Biology Laboratory, Wildlife Institute of India, Dehradoon - 248 001; 2 Amity Institute of Advanced Forensic Science Research and Training, Noida, India

**Keywords:** DNA, hair evidence, microscopy, keratin, wildlife forensics

## Abstract

India is known for its rich biodiversity, with a wide variety of wild floral and faunal species. This wildlife treasure of ours faces the threat of extinction due to rampant poaching and illegal trade. With most of the wildlife offence cases related to mammals having hair as physical evidence, it becomes imperative to use this evidence in the best possible way for wildlife crime investigation. We discuss the value of hair evidence with special reference to species characterization/identification using microscopic hair characteristics, keratin patterns, and mitochondrial DNA typing. The relevance of the techniques with respect to Indian scenario is specially taken care of and microscopic hair characteristics of one of the highly endangered species along with its keratin pattern are described. Finally, the use of mitochondrial DNA for species identification is also discussed.

## INTRODUCTION

India is identified as one of the 12 mega-biodiversity nations, having 8% of the world′s biodiversity with 60% of world′s tiger, 50% of Asian elephants, 70% of Asian rhinos, and the only population of Asiatic lion in wild.[1] India further has the rare blend of Palaearctic, Oriental, and Afrotropical fauna[2] with over 400 species of mammals.[3] We face the threat of losing all this precious wildlife due the rampant poaching and growing illegal wildlife trade.

Mankind has been exploiting wildlife and natural resources since times immemorial for food, clothes, medicine, pleasure, and profit, but the commercial exploitation in the recent years decimated some of the species to the verge of extinction.[4] Thus, conservation of wild species today is a major challenge due to persistent illegal trade in wildlife parts and products.[4] Annual international trade in wildlife and its products has been estimated to be of approximately US $20 billion, of which illegal trade in wildlife alone is of US $5 billion and in economic terms it ranks second after the drugs.[1] Most of the trade is highly clandestine and hence it is very difficult to provide correct estimates about the magnanimity of the trade and probably the actual figures may be very high.

Wildlife in India is protected under the Wildlife (Protection) Act 1972 of India. All the animals listed under the schedules I, II, III, and IV of the Wildlife (Protection) Act 1972 are protected and poaching amounts a punishable offence under this act. Further, India is also a party to *Convention on International Trade in Endangered Species* (CITES). However, despite such strong legislation in place, poaching has still increased rampantly over the years, which has decimated the population of several wild species and has brought their numbers to an alarmingly low level. It has been observed that in most of the poaching cases the conviction rate is very low. Annon[5] attributes this to the lack of adequate species characterization and identification techniques from the confiscated articles. In order to prosecute a person under the Wildlife Protection laws, it becomes necessary to identify the species of the poached animal; which is not possible due to the lack of know-how, database, and expertise in the field. The problem gets compounded even more when finished products need to identified and further aggravating the situation, there are fakes floating in the market. Thus, it becomes imperative to develop techniques for species characterization from wildlife articles confiscated under wildlife protection laws.

Mammals form one of the largest groups of poached species and in most of the cases related to mammalian poaching, hair is invariably found as physical evidence. Wildlife forensic laboratory at the Wildlife Institute of India, Dehradoon, on an average annually receives exhibits from about hundred wildlife offence cases for species identification. Out these, nearly 50–60% of the cases revolve around the identification of species from hair.

The value of hair as a physical evidence is well appreciated in crime investigation. Further, hair by virtue of its chemical composition resists degradation by environmental factors and enzymatic activities to fairly good extent. The morphology and keratin (protein) of hair can be used for species identification, and in addition to these it contains the nuclear DNA (when root is present) and mitochondrial DNA of an individual. Thus, hair can be used to serve the purpose of species identification with help of morphometric, protein and DNA-based techniques.

Microscopic hair characteristics of hair have been used for species characterization by Hausman,[6‐8] Mathiak,[9] Mayer,[10] Adorjan and Kolenosky,[11] Joslin,[12] Brunner and Coman,[13] Moore *et al*[14] and Teerink.[15] Further, the polymorphism of hair keratins is well established and has been used for species identification. After immunoglobulins, keratins are perhaps the most heterogeneous type of proteins. Although several explanations for the same have been given, but most likely it is due to multiple gene loci coding for the keratins.[16] There might also be considerable differences between species, subspecies, varieties, and breeds and perhaps between the individuals of the same species.[17] Prior to advent of DNA technology, protein methods were very frequently used for purpose of species and individual identification in crime investigation. Some workers like Caracedo *et al*,[18] advocated keratin profiling for identification of species instead of microscopic hair characteristics.

On going through published literature, it becomes apparent that keratin patterns have been immensely used for species characterization. Budowle and Acton[19] reported electrophoretic detection of proteins in hair (essentially keratin and keratin associated proteins) samples and also reported its variation in their electrophoretic patterns. Similarly, Marshall[20] worked on analysis of the proteins from single wool fiber by 2-dimensional polyacrylamide gel electrophoresis (2D-PAGE). Again, Marshall and Gillespie[21] compared the keratin profiles/patterns of human hair by using 2D-PAGE. They were able to discriminate between hair samples of individuals based on the keratin patterns. Marshall,[22] again carried out studies on characterization of human nail proteins (essentially keratin and keratin associated proteins) using electrophoretic methods. Methods for species identification from hair, through keratin patterns have been discussed by Marshall and Gillespie.[23] Carracedo *et al*,[24] worked on iso-electric focusing (IEF) of hair keratins and its subsequent silver staining for obtaining keratin patterns. Carracedo *et al*, [18] studied the keratin patterns from hair of 97 animals from six orders, i.e. carinovora (*Canis lupus, Panthera tigris, Meles meles, etc*.), primates (*Homo sapiens, Papio papio, Pan troglodytes, Macaca irus*, etc.), artiodacytla (*Sus scrofa, Lama glama, Dama dama*, etc.), perissodactyla, lagomorpha, and rodentia with IEF followed by silver staining. Later, Butler *et al*,[17] studied rhinoceros keratins by IEF for five species of Rhinos, viz. *Rhinoceros unicornis, Rhinoceros sondaicus, Didermocerus sumatrensis, Diceros bicornis, Eratherium simum*). Butler[17] also studied keratins from the hair samples of mule deer (*Odecoileus hemionous*), white tailed deer (*Odecoileus virginianus*), cat (*Felis catus*), moose (*Alces alces*), elk (*Cervus elaphusnelsoni*), antelope, woodland caribou (*Rangifer terandus*), raccoon, goat (*Capra hircus*)and from feather samples of parrot and goose and shaving of elephant tusk ivory (*Loxodonta africana*and*Elephas maximus*), bovine and rhinoceros horn (*Rhinoceros unicornis*), and deer antler. Rodriguez *et al*,[25] studied the changes in electrophoretic pattern of cosmetically treated hair with the help of IEF correlation with sodium dodecyl sulphate polyacrylamide gel electrophoresis (SDS-PAGE). Folin and Contiero[26,27] carried out electrophoretic analysis of mammalian keratins and some nonhuman primates with IEF to serve taxonomic purposes.

Although the microscopic hair characteristics and keratin patterns can serve the purpose of species identification, however it has certain limitations, which have already been mentioned; especially that an individual can produce variety of hair samples with different structures and still further hair structure can be affected by environmental factors in a longer run. Although, hair samples being composed of keratin are much more stable than other soft tissues, but they do get affected by environmental factors slowly, showing degradation and thereby showing alterations in the protein profiles. Therefore, it becomes imperative to use DNA-based techniques to deal wildlife offence cases keeping in view their high sensitivity, precision, consistency, and reproducibility. Species identification from DNA has quite a few approaches, but the commonly used approaches are restriction fragment length polymorphism (RFLP), polymerase chain reaction-restriction fragment length polymorphism (PCR-RFLP), randomly amplified polymorphic DNA (RAPD), and forensically informative nucleotide sequencing (FINS). In wildlife offence case, the latter is the best as there is very little control over quality and quantity of DNA obtained. Hair samples obtained in wildlife offence cases present a peculiar problem as they mostly lack intact roots (follicular cells) as such there is little possibility of obtaining high molecular weight DNA. Thus, the use of techniques like RAPD that require high molecular weight DNA cannot be used. FINS is the most useful DNA-based technique that can be put to use in species identification from hair. FINS involves amplification and sequencing of a segment of a conserved mitochondrial gene (Cytochrome b, 16s rRNA, 12s rRNA, etc.). The sequence in then later matched with reference sequence database available with NCBI/GenBank (National Center for Biotechnology Information: website www.ncbi.nlm.nih.gov) through Basic Local Alignment Search Tool (BLAST). Further confirmation can be made by actually sequencing a reference sample and matching the questioned sample with it.

These techniques have been exploited to a good limit for species identification around the globe, however in the Indian context very little work has been carried out on species characterization from hair and most of the studies are centered on determining the prey composition of carnivores using microscopic hair characteristics. The work is basically related to study of prey composition of carnivore species like lion.[12] Koppikar *et al*,[28,29] worked on identification of hairs of some Indian mammals. De *et al*,[30‐32] reported hair characteristics of some primate (*genus Panthera*) and Mongoose species (*genus Herpestes*). Similarly, Chakraborty *et al*,[33,34] reported hair characteristics of three lesser cats found in India and of cheetah *Acinonyx jubatus venaticus* (Griffith) and lesser panda, *Ailurus fulgens*. A scanning electron microscopy (SEM) study on cuticular pattern of guard hair of Tibetan antelope (*Pantholops hodgsoni*) was carried out by Bahuguna and Mukherjee[35] for species characterization from a forensic view point. Sahajpal & Goyal[36‐39] highlighted the usefulness of the microscopic hair characteristics for species identification for dealing wildlife offence cases in India. Sahajpal *et al*[39] reported the guard hair characteristics of four Indian bear species and mongoose species for species identification and dealing poaching cases related to bears and mongooses. With regard to studies on keratin patterns for species characterization in India, only one study has been reported on electrophoretic characterization of keratins by Sahajpal and Goyal,[37] which deals with use of SDS-PAGE to identify Shahoosh (*Pantholops hodgsoni*), Pashmina (*Capra hircus*), and Angora (*Oryctolagus cunniculus*). In DNA scenario, some work has been done in India. Girish *et al*,[40] used mitochondrial 12S rRNA gene for species characterization from meat products. They amplified about 400 base pair (approx.) fragments of 12S rRNA gene and sequenced it for species identification. Verma and Singh[41] have shown the usefulness of universal primers for species identification from biological samples in illegal trade. Similarly, Guha and Kashyap[42] and Guha and Chattopadhyay[43] used FINS for detection of lizard species in case of food poisoning. However, there was a great lack of mitochondrial DNA sequence data for Indian wild fauna as such many times was not possible to make proper comparisons.

Therefore, it was the need of the hour to refine the existing techniques on species identification from hair by adding new analytical parameters and to develop new and more precise techniques for species identification and to generate a scientific database. Keeping this in view, initiative was taken at the Wildlife Institute of India to develop techniques and maintain database for species characterization from hair evidence.

## MATERIALS AND METHODS

### Microscopic studies

Microscopic hair characteristics (with light microscopy and SEM) for some of the most endangered species (*n* =57) listed under Schedules I–IV of the Indian Wildlife (Protection) Act 1972 were[44] studied. Five samples for each species were studied and these were obtained from the reference collection of the wildlife forensic laboratory of the Wildlife Institute of India. All the hairs had been collected from the mid-dorsal region of the animals. The microscopic hair characteristics were determined according to the protocols provided by Sahajpal and Goyal.[36] Classification given by Brunner and Coman[13] was used to describe the hair characteristics

### Keratin pattern studies

A study on keratin patterns was undertaken to develop a database for some of the most endangered species like *Pantholops hodgsonii* (animal from which shahtoosh wool is obtained) using SDS-PAGE. A protocol with following steps standardized and followed for the studies.

### Sample preparation

Five samples of each species were taken from the reference collection of Wildlife Forensic laboratory, Wildlife Institute of India. The samples were washed thoroughly in distilled water and alcohol to remove the dirt. Washed hair samples were chopped into small pieces.

### Preparation of soluble proteins

Ten milligrams of chopped hair sample were taken for each species which was extracted with the help of 200 µl extraction buffer comprising of 12 M urea, 74 mM trizma base, 78 mM dithiothreitol (DTT) as described by Marshall and Gillespie[23] but without carboxymethylation. The extraction was carried over a period of 48 h under inert atmosphere of nitrogen or by covering the reaction mixture with mineral oil to avoid contact with air. Twenty five microliters of this extract were treated with 5 µl of 0.1 M DTT solution and incubated at 25°C for 10 min. To 10 µ1 of this mixture 10 µl of sample buffer containing SDS was added and extracted proteins are denatured by heating at 96°C for 5 min.

### Running of SDS-PAGE

Vertical SDS-PAGE was carried on Hoefer S.E-260 (10 × 10.5 cm gel) using Schagger and Von and gel system at a constant voltage of 50 V and current of 20 mA by loading 5 µl of heat denatured extract of each sample. 2.5 µl of low molecular weight calibration kit (Amersham Biosciences) was also run along the samples. The reagents (SIGMA mol bio grade) required for gel preparation and running are as follows:

1. Separating gel acrylamide: 48 g of acrylamide, 3 g of N,N'-methylene bisacrylamide in 100 ml of deionized water.
2. Spacer gel acrylamide: 48 g of acrylamide, 1.5 g of N,N'-methylene bisacrylamide in 100 ml of deionized water.
3. Stacking gel acrylamide: 30 g of acrylamide 0.8 g of N,N'-methylene bisacrylamide in 100 ml of deionized water.
4. Separating gel buffer: 3 M Tris–HCl, 0.3% SDS (pH 8.9).
5. Stacking gel buffer: 1 M Tris–HCl, (pH 6.8).
6. Cathode buffer (10X): 1 M Tris base, 1 M Glycine, 1% SDS.
7. Anode buffer (10X): 2 M Tris–HCl (pH 8.9).
8. 10% ammonium persulphate (APS)
9. 0.2 M tetrasodium EDTA.
10. Glycerol.

### Casting of gels

1. Separating gel: 3.35 ml H_2_O, 5 ml separating gel buffer, 5 ml separating gel acrylamide,1.6 ml glycerol, 5 µl TEMED, and 50 µl of 10% APS.
2. Spacer gel: 3.45 ml H_2_O, 2.5 ml separating gel buffer, 1.5 ml spacer gel acrylamide, 2.5 µl TEMED and 25 µl of APS.
3. Separating gel: 5.15 ml H_2_O, 0.95 ml stacking gel buffer, 1.25 ml stacking gel acrylamide, 75 ml of EDTA, 3.35 µl TEMED, and 75 µl of APS.

### Staining, photography and determination of molecular weights

Coomassie (0.1% coomassie brilliant blue R250 in 50 ml of methanol, 10% glacial acetic acid) staining was done overnight and the stained gels were photographed using a gel doc system (UVP) and the molecular weights of the bands for each species were calculated using Gene profiler software.

### Mitochondrial DNA studies

#### DNA isolation

DNA isolation from hair of wildlife species becomes a challenge as mostly the samples may be old and lacking roots. Qiagen DNeasy^®^ tissue kit (Qiagen Germany) was used for DNA isolation with slight modification, i.e. 20 μl of 100 mM dithiothreitol (DTT) was added to the 180 μl lysis buffer (ATL).

#### PCR amplification

The PCR amplifications were carried out using the following primers which amplified cytochrome b (398 bp), 12s rRNA (400 bp), and 16s rRNA (540 bp) gene segments.

1. Cytochrome b 398 bp.[41]
  Primer forward: 5′-TACCATGAGGACAAATATCATTCTG-3′
  Primer reverse: 5′-CCTCCTAGTTTGTTAGGGATTGATCG-3′
2. 12 s rRNA 400 bp.[40]
  Primer forward: 5′-CAAACTGGGATTAGATACCCCACTAT-3′
  Primer reverse: 5′-GAGGGTGACGGGCGGTGTGT-3′
3. 16 s rRNA 540 bp.[45]
  Primer forward: 5′-CGCCTGTTTATCAAAAACAT-3′
  Primer reverse: 5′-CTCCGGTTTGAACTCAGATC-3′

#### DNA sequencing

The DNA sequencing was carried out only with forward primers of the universal primers selected for analysis. Sequencing was performed according to standard protocol provided for Big dye terminator kit^®^ V 3.1 (ABI Prism 3130 Genetic analyzer). The reaction products were cleaned with help of Qiagen DyeEx^®^ spin kit to remove unincorporated ddNTPs. The data was analyzed with ABI Sequencing Analysis V 5.2 software.

## RESULTS

### Microscopic studies

**Figure 1:**
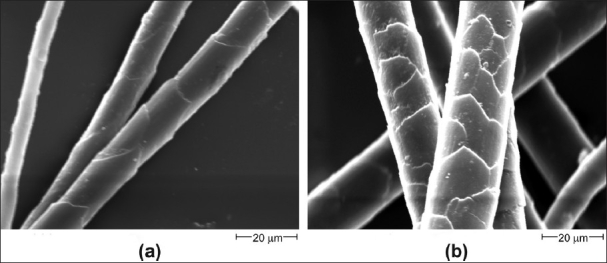
Scanning electron micrograph of cuticle of (a) Shahtoosh wool (*Pantholops hodgsonii*) and (b) Pashmina (*Capra hircus*) wool

**Figure 2:**
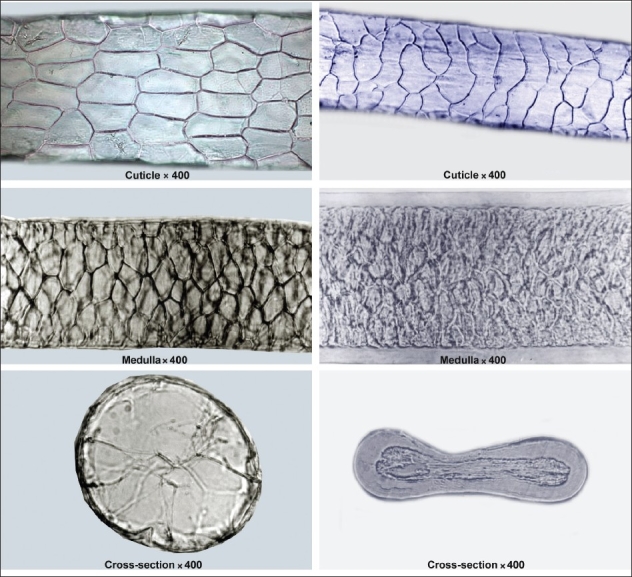
Microscopic hair characteristics (cuticle, medulla and cross-section) of *Pantholops hodgsonii* (left side) and *Capra hircus* (right side)

Species could be characterized/identified using a combination of microscopic hair characteristics like cuticular scale type, pattern, medulla type, medullary index values, and cross-section shapes. Microscopic hair characteristic (cuticle) of the wool of Tibetan antelope (*Pantholops hodgsonii*), a highly endangered and protected species from which the highly prized shahtoosh wool is obtained in comparison to microscopic hair characteristics (cuticle) of Pashmina goat (*Capra hircus*) a domesticated species of Himalayas (Ladakh) are shown in Figure 1. Similarly, the cuticular, medullar, and cross-sectional structure of the guard hair of Tibetan antelope and Pashmina goat is shown in Figure 2. The observations for microscopic hair characteristics are given in Table 1. It becomes apparent from Figures 1 and 2 and Table 1 that the two species can be distinguished from each other easily by their microscopic hair characteristics. The cuticular patterns for two species are very distinct with *Pantholops hodgsonii* showing regular mosaic pattern and *Capra hircus* showing a regular wave pattern. Similarly, it is possible to distinguish between other species studied based on the combination of microscopic hair characteristics.

**Table 1:** Microscopic hair characteristics of *Pantholops hodgsonii* and *Capra hircus*

Species	Cuticular pattern	Medulla type	Medullary index	Crosssection
*Pantholops hodgsonii*	Regular mosaic	Wide medulla lattice	0.96‐0.98	Circular	
*Capra hircus*	Regular wave	Wide medulla	0.76‐0.82	Oblong to dumb-bell	

### Keratin pattern studies

**Figure 3:**
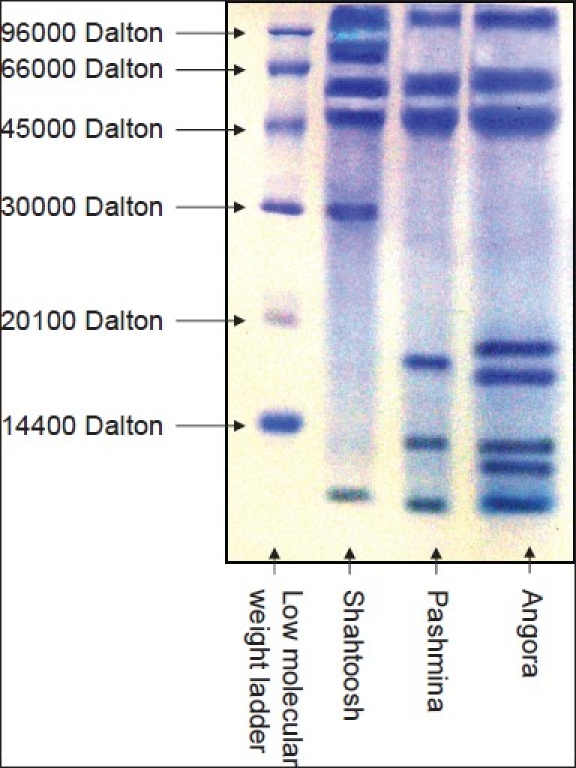
Keratin pattern of Shatoosh, Pashmina and Angora wool using SDS-PAGE (Sodium dodecyl sulphate polyacrylamide gel electrophoresis) with coomassie staining

The keratin patterns of Shahtoosh, Pashmina, and Angora rabbit wool are shown in Figure 3. The molecular weights of the separated keratins are given in Table 2. It is clear from Figure 3 that keratin patterns can be used to distinguish between the species studied. Similar differences were observed in all the other species studied.

**Table 2:** Molecular weight of the separated keratins (in daltons) for the three species using SDS-PAGE

Shahtoosh	Pashmina	Angora
113152	112363	113152
77531	58571	60779
57902	46553	47235
47007	17535	18461
29697	13525	16842
11461	11132	13435
(----)	(----)	12562
(----)	(----)	11132

### DNA studies

**Figure 4:**
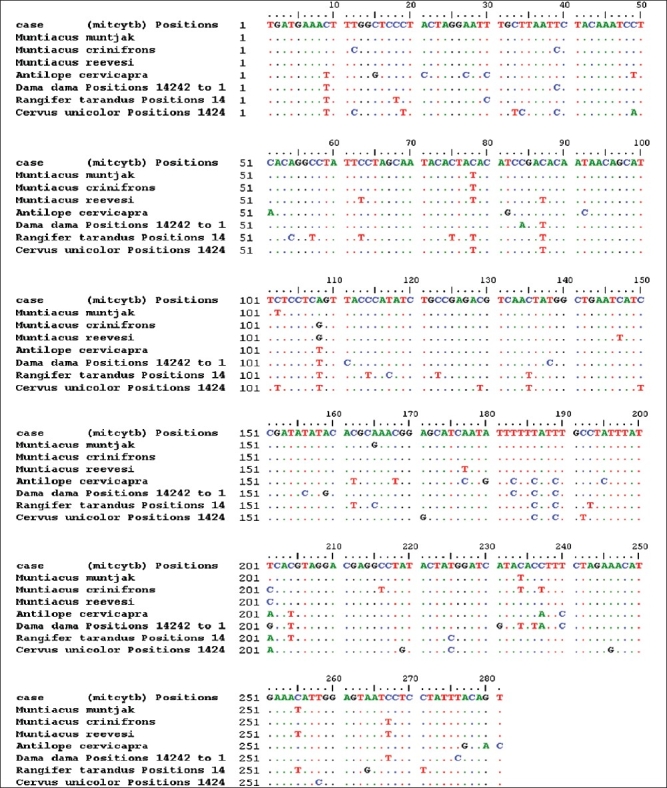
Mitochondrial cytochrome b DNA sequence of a wildlife offence case exhibit aligned with sequences of other species

**Figure 5:**
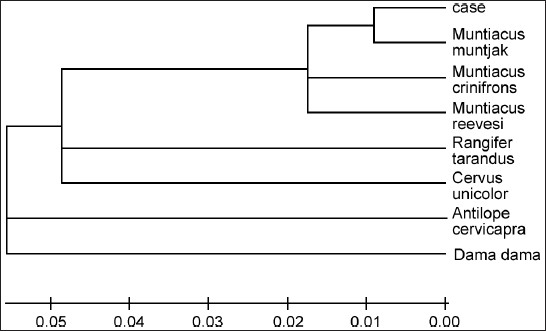
Neighbour joining tree (NJ tree) plotted using MEGA for the aligned mitochondrial DNA sequences of case sample and other species. The case sample goes with *Mutiacus muntjac*

Extraction of DNA from hair samples was possible by following the modified Qiagen kit protocol. The DNA extracted by this protocol was successfully amplified using the selected primer and sequenced. The sequences have been contributed to GenBank/NCBI and are accessible to researchers working in this field. These can be used to identify species from unknown samples through DNA using the BLAST tool of NCBI. Figures 4 and 5 show comparison of mitochondrial DNA sequence (cytochrome b) of a questioned hair sample with some of the sequences of likely species. From Figure 4 it is clear that there is about 99% similarity in bases of questioned sample and barking deer (*Muntiacus muntjac*) from the neighbor joining tree (NJ tree) generated using MEGA (Molecular Evolutionary version 4.0.).[46] It is clear that the suspected sample matches with *Muntiacus muntjak*, which is a protected species under the Indian Wildlife (Protection) Act 1972.

## CONCLUSION

It is apparent the hair evidence is of great value in wildlife crime investigation in India. The microscopic hair characteristics corroborated with keratin pattern studies provide a sound basis for species identification. The DNA studies further strengthen the science of species characterization from hair. The data generated from this study will be of immense significance not only for routine forensic analysis for species identification from hair but also facilitate the successful implementation of Indian Wildlife (Protection) Act 1972. This will lead to conviction of poachers under the law and conservation of the wild fauna and flora for coming generations of mankind.
